# In this month’s Bulletin

**DOI:** 10.2471/BLT.26.000926

**Published:** 2026-09-01

In the editorial section, Colette SA Wesonga and Benard W Kulohoma (586) debate ways to address health financing pressures and donor transition in Kenya.

## Bangladesh

### Quantification of antibiotics used in livestock.

SM Sabrina Yesmin et al. (613–621) analyse data on veterinary use.

## Madagascar

### Paediatric schistosomiasis prevention

Valentina Marchese (601–612) study the feasibility of using praziquantel.

## United Republic of Tanzania 

### Counting maternal deaths

Ahmad Mohamed Makuwani et al. (631–639) review the factors affecting mortality estimates.

## WHO South-East Asia Region

### Penicillin for syphilis and rheumatic heart disease.

Gereltuya Dorj et al. (589–600) assess the market landscape.

## Global

### Updates to the *International statistical classification of diseases and related health problems*

Jonathan ML White et al. (622–630) explain how the medical and scientific advisory committee reviews proposals.

### Antimicrobial resistance surveillance 

Cyril Buhler et al. (640–645) present a suite of laboratory tools. 

### Occupational heat exposure

Xin Zhou et al. (646–648) point to a need for better worker protection policies.

### Healthy Life Trajectories Initiative

Michelle Pentecost (649–650) highlights evidence from a cohort study.

### A transdisciplinary research agenda 

Anne-Sophie Jung et al. (651–653) reframe health system resilience.

### Air pollution affects environmental, animal and human health.

David Rojas-Rueda (654–656) argues for the inclusion of air pollution in One Health strategies.

